# Environmental Crisis and the Emergence of the Oropouche: A Potential Public Health Problem

**DOI:** 10.1590/0037-8682-0295-2024

**Published:** 2024-12-16

**Authors:** Marina dos Santos Barreto, Ronaldy Santana Santos, Lucas Alves da Mota Santana, Rajiv Gandhi Gopalsamy, Govindasamy Hariharan, Bernardo Ferreira Brasileiro, Ricardo Queiroz Gurgel, Dalmo Correia, Cleverson Luciano Trento, Lysandro Pinto Borges

**Affiliations:** 1 Universidade Federal de Sergipe, Departamento de Farmácia, São Cristóvão, SE, Brasil.; 2 Universidade Federal de Sergipe, Departamento de Odontologia, Aracaju, SE, Brasil.; 3 Rajagiri College of Social Sciences, Division of Phytochemistry and Drug Design, Department of Biosciences, Kochi, Kerala, India.; 4 Bharata Mata College (Autonomous), Department of Chemistry, Thrikkakara, Kochi, Kerala, India.; 5 Southwest Florida Oral and Facial Surgery, Oral and Maxillofacial Surgeon, Private Practice, Fort Myers, FL, USA.; 6 Universidade Federal de Sergipe, Departamento de Medicina, Aracaju, SE, Brasil.; 7 Universidade Federal de Sergipe, Programa de Pós-Graduação em Ciências da Saúde, Aracaju, SE, Brasil.; 8 Universidade Tiradentes, Aracaju, SE, Brasil.; 9 Universidade de São Paulo, Departamento de Imunologia, Instituto de Ciências Biomédicas, São Paulo, SP, Brasil.

Brazil is a South American country where arboviruses such as dengue, Zika, Chikungunya, and yellow fever are endemic. There are also reports of sporadic outbreaks of viruses such as the Oropouche virus (OROV), which is an *Orthobunyavirus* ^1^. Although it has not received much attention, this virus was responsible for more than 7,000 documented cases in the country by July 2024, an eight-fold increase compared to the entire previous year, which saw just over 800 cases^2^. Recently, the first fetal death from the vertical transmission of OROV fever worldwide was documented^2^. The case occurred in the northeastern city of Pernambuco. The mother was 28 years old and was in her 30th week of pregnancy. A second suspected case in the same state that resulted in a miscarriage is being investigated^2^. Health authorities and the general population have overlooked this disease, in a similar manner to the Zika virus prior to the congenital syndrome caused by Zika raising levels of concern^2^. Events like this emphasize the importance of closely monitoring the public health impact of viruses like OROV. In this letter, we draw attention to OROV fever, which has been causing serious consequences in the Brazilian population, characterizing it as a public health problem. In addition to the common symptoms, such as fever, headache, nausea, vomiting, and dizziness, more intense symptoms, such as severe headache, balance dysfunction, and photophobia can occur^3^. In the most severe cases, neurological damage such as brain inflammation, dizziness, and lethargy can occur, as well as miscarriages and congenital damage such as microcephaly, which are possibly associated with the virus^3^. Recently, OROV genetic material has been identified in the umbilical cord, brain, liver, kidneys, lungs, heart, and spleen of a fetus exposed to vertical transmission^3^. This finding promotes a greater understanding of the importance of research on and prevention of this virus. 

Environmental imbalance is an important factor contributing to the spread of arboviruses such as OROV. The role of the ecological crisis and rampant deforestation in the spread of viral agents and the emergence of pandemics have gained prominence following the COVID-19 pandemic^4^. According to Tollefson (2020)^4^, the destruction of wildlife habitats favors the growth of species more likely to survive, such as rats and bats. These species are more likely to be hosts for pathogens that are potentially dangerous to human health. In addition to deforestation, activities such as hunting, and the illegal sale, purchase, and consumption of wild animals can contribute to pathogens reaching the human body and causing infections^4^. 

Climate change is also a progressive factor linked to the spread of potentially dangerous pathogens^5^. In recent years, the global temperature has risen significantly; 2023 is the hottest year ever recorded, and 2024 may have temperatures that could equal or break this record^6^. A previous study reported that an increase in temperature can affect the genetic diversity of viral populations in a strain-dependent manner. This suggests that temperature is an ecological driver of mixed (virus-virus) infections, with an effect on the genetic diversity of individual viruses^7^. Another study found that an increase in temperature led to a variation in the viral load of RNA viruses; however, the average viral load did not change^8^. The authors point out that these results suggest that with increasing temperature, more-susceptible species become even more susceptible, and less-susceptible species become even less susceptible. Thus, changes in global temperature can lead to changes in hosts and pathogens, thereby generating new opportunities for transmission. However, these changes can either increase or decrease the ability of the pathogen to reach its host^8^. Liam et al. (2023)^9^ used a forecasting method to assess the epidemiology of SARS-CoV-2 infections. The authors suggested that the COVID-19 outbreak in the summer of 2022 was consistent with heat waves and that approximately 70% of COVID-19 cases could have been prevented if these waves were avoided^9^. Additionally, viral vectors may find more favorable environments for their development as temperatures and global geographical areas change because of the climate crisis^5^. However, some questions regarding how temperature affects virulence and viral pathogenicity remain unanswered and require further study^7^. Since the 1990s, researchers have demonstrated the possible influence of changes in landscapes and fauna on the resurgence of eradicated zoonotic diseases^10^ ^,^ ^11^. 

*Culicoides paraensis*, known as the gunpowder mosquito, is the main vector responsible for OROV’s urban disease cycle. Wild species such as sloths, non-human primates, and possibly birds and rodents are the hosts responsible for the wild cycle^2^. Controlling the spread of the virus among vectors or preventing human contact with their habitats could halt viral spread. 

Despite its impact, OROV fever remains an overlooked disease because its symptoms resemble those of dengue fever (fever, nausea, vomiting, and headache)^2^. To date, there are no treatments for OROV fever or vaccines to prevent infection or illness. Development of the dengue vaccine took more than 75 years, and the vaccines developed to date still have limitations for some population groups^12^. In Brazil, the first dengue epidemic was recorded in 1981-1982 and has been documented continuously since then, resulting in more than 9,000 deaths between 2000 and 2023^12^. The dengue vaccine has only recently been made available free of charge by the country's Unified Health System (2023)^12^. Such deaths could have been minimized and possibly avoided if necessary attention had been paid to the disease from the beginning of the epidemic, with support for the development of an effective vaccine. Although there are no vaccines or treatments for OROV, infected people should rest at home, treat their symptoms, and seek medical attention. Preventive measures to stop this spread include avoiding areas where it occurs, minimizing exposure to vectors, covering up with clothing and repellents, and using fine-mesh screens on doors and windows^2^. 

In this letter, we draw attention to the occurrence of OROV fever in Brazil and its serious health consequences in terms of congenital and cognitive problems. This disease requires attention, which is why we highlight studies of the viral genome, as well as government interventions aimed at health education, vaccine development, and the containment of vectors in urban areas. Research into larvicidal compounds is required and encouraged. However, we emphasize the importance of taking effective action to reduce deforestation and illegal hunting, which contribute to the spread of pathogenic viruses such as OROV. Therefore, there is an urgent need to develop vaccines to reduce the transmission and complications of OROV, as well as to minimize its impact on children.
